# Comparative evaluation of triplet antiemetic schedule versus doublet antiemetic schedule in chemotherapy-induced emesis in head and neck cancer patients

**DOI:** 10.3332/ecancer.2015.567

**Published:** 2015-08-25

**Authors:** Pulkit Kaushal, Rajeev Atri, Abhishek Soni, Vivek Kaushal

**Affiliations:** 1 Department of Psychiatry, Seth GS Medical College and KEM Hospital, Mumbai 400012, Maharashtra, India; 2 Department of Radiotherapy, Pt. B. D. Sharma Post Graduate Institute of Medical Sciences, Rohtak 124001, Haryana, India

**Keywords:** aprepitant, antiemetic, CINV (chemotherapy-induced nausea and vomiting), head and neck cancer, palonosetron

## Abstract

**Purpose:**

To compare the antiemetic combination of palonosetron, dexamethasone, and aprepitant (PDA) with antiemetic combination of ondansetron and dexamethasone (OD) in head and neck cancer patients receiving docetaxel, carboplatin, and 5-FU based chemotherapy.

**Methods:**

Sixty previously untreated patients were randomly divided into two groups of thirty patients each. The PDA group received a combination of palonosetron 0.25 mg intravenously (IV), dexamethasone 12 mg IV, and capsule aprepitant per oral. OD group received ondansetron 16 mg IV, and dexamethasone 12 mg IV for emesis control. The primary objective was to compare the efficacy of two antiemetic schedules for preventing acute and delayed CINV (chemotherapy-induced nausea and vomiting). The primary efficacy end point was complete response (CR).

**Results:**

All the patients tolerated both schedules well. The antiemetic response for acute emesis (first 24 hours) in PDA versus OD group was: CR was 86.7 versus 60%. For delayed emesis (from day 2–5) in PDA versus OD group CR was 83.3 versus 53.3%. The intensity of acute nausea (first 24 hours) in PDA versus OD group was: no nausea–70 versus 46.6%. The intensity of delayed nausea (from day 2–5) in PDA versus OD was: no nausea–76.6 versus 43.3%. The CR to both acute and delayed emesis (no vomiting from day 1–5) in PDA versus OD group was 83.3 versus 53.3% (p < 0.05, significant). The CR to nausea (no nausea from day 1–5) in PDA versus OD group was 70 versus 43.3% (p < 0.05, significant).

**Conclusion:**

Although both the schedules were tolerated well, the PDA schedule (palonosetron, aprepitant, and dexamethasone) was significantly better than the OD schedule (ondansetron and dexamethasone) in controlling cancer CINV in the acute as well as delayed phases.

## Introduction

CINV is one of the most unpleasant side effects of cancer treatments experienced by cancer patients [1, 2, 3]. Chemotherapy-induced emesis can cause anorexia, nutritional deficiency, metabolic imbalances, altered mental status, degeneration of self care, wound dehiscence, and esophageal tear [4]. Furthermore, its prolonged manifestation may reduce patients’ quality of life (QOL) [5]. It may also lead to noncompliance of anticancer therapy with premature discontinuation [4]. As chemotherapy in a curative and palliative way is the key mode of treating cancer; preventing, minimising, and treating CINV have become an indispensable aspect of cancer chemotherapy. Although significant progress has been made in the treatment of CINV, patients undergoing chemotherapy continue to report that this side effect is persistent and distressing [4, 6, 7].

Nausea, vomiting, and retching, although related, are actually three distinct symptoms and often go hand-in-hand [1, 8, 9]. Chemotherapy-induced emesis can be classified as acute (first 24 hours) and delayed (day 2–5) [1, 10–12]. The emetic process, triggered by chemotherapeutic agents, involves a complex network of neuroanatomical and peripheral centres, neurotransmitters, and receptors [1]. The three main neurotransmitters are serotonin/5-hydroxytryptamine (5-HT), substance P (SP), and dopamine. The receptors associated with 5-HT and SP are 5-hydroxytryptamine (5-HT3) and neurokinin-1 (NK-1) respectively [13].

Without antiemetic prophylaxis, the CINV incidence varies from 30–90% for acute phase and 28–50% for delayed phase post moderately emetogenic chemotherapy (MEC) [14–17]. Prevention and control of acute phase CINV is linked with decreased incidence of delayed phase CINV [15]. Similarly, prevention and control of CINV in the first chemotherapy cycle leads to decreased CINV incidence in the subsequent chemotherapy cycles [18, 19]. As 5-HT3 receptors are involved in acute phase CINV, two drug therapy with 5-HT3 receptor antagonists and dexamethasone are effective in acute phase CINV [18, 20, 21]. Substance P and neurokinin-1 receptors are involved in delayed phase CINV, thus, triple drug therapy with aprepitant is used, which is effective in acute as well as delayed phase of CINV [18, 21–23]. Mostly, delayed phase CINV is underestimated by the clinicians [18, 21]. Furthermore QOL deteriorates more with delayed phase CINV, suggesting aprepitant addition for CINV control [19, 24, 25]. In Indian scenario, data suggesting aprepitant use is very limited. Therefore, the present study was undertaken to compare the efficacy, tolerability, and side effects of newer triplet antiemetic combination of palonosetron, dexamethasone, and aprepitant with the conventional doublet antiemetic combination of ondansetron and dexamethasone in patients receiving MEC for head and neck cancer.

## Material and methods

### Patients

This open labeled, prospective, randomised trial was conducted on sixty previously untreated histopathologically-proven patients of squamous cell carcinoma of head and neck, attending the Department of Radiotherapy, Pt. Bhagwat Dayal Sharma Post Graduate Institute of Medical Sciences, Rohtak, for definitive treatment. The tumour staging was done using the American Joint Committee on Cancer (AJCC) TNM staging system 2010 [26].

The eligibility criteria were: Karnofsky Performance Status ≥70, normal complete haemogram, and blood biochemistry within normal limits. Excluded from the study were patients having: distant metastases, pregnant or lactating females, history of allergy to ondansetron, palonosetron, or aprepitant, receipt of chemotherapy during the seven days before study drug administration, any associated medical condition causing nausea/vomiting (e.g. renal, liver, or heart disease). All patients provided written informed consent.

### Study design and treatments

The combination chemotherapy schedule in all patients consisted of chemotherapy with docetaxel 60 mg/m^2^ intravenously (IV), carboplatin 300 mg/m^2^ IV, and 5-FU (5-Fluorouracil) 600 mg/m^2^ IV. Planned chemotherapy was to be ≤4 hours. At least seven days before chemotherapy administration, patients discontinued antiemetics and systemic corticosteroids (including dexamethasone). The patients were divided randomly in two groups of thirty patients each. The study group was administered with PDA antiemetic schedule (palonosetron plus dexamethasone plus aprepitant) and the control group was administered with OD schedule (ondansetron plus dexamethasone) as per the Table 1. The study was approved by the Institutional Ethical Committee and the Indian Council of Medical Research with reference number 2010-00047 Haryana.

### Objectives and efficacy evaluations

The primary objective was to compare the efficacy of two antiemetic schedules i.e. palonosetron plus dexamethasone plus aprepitant versus ondansetron plus dexamethasone in patients receiving MEC for head and neck cancer. The secondary objective was to determine the tolerability and side effects of the above two antiemetic schedules. The primary efficacy end point was the CR (no emetic episodes and no use of rescue medications) during the acute (0–24 hours) and delayed (24–120 hours) phases after chemotherapy. Secondary end points included safety with CR over the entire (0–120 hours) period.

The antiemetic effects were evaluated by recording frequency of vomiting (acute and delayed), and intensity of nausea. The frequency of vomiting was assessed as the proportion of patients with emesis in the acute (day 1) and delayed (days 2–5) phases after chemotherapy. A single emetic episode was defined as emesis separated by less than a five minute interval. Delayed response was graded depending upon the worst observation from day 2–5. Nausea was defined by a patient’s report of a feeling in the stomach that he/she may vomit.

The reporting and recording of acute nausea and vomiting were performed at the hospital, while the intensity and frequency of delayed nausea and vomiting were recorded by the relatives of the patients. Those relatives were explained about the detailed procedure. They were instructed to record the frequency of emetic episodes on the protocols provided, which were then collected at the next hospital visit. For intensity evaluation, the patients were instructed to place a finger at a point on the descriptive ordinal scale (DS), depending on the intensity of nausea felt by them. [13] Patients were given instructions to document the number of emetic episodes and nausea severity during the five day observation period after the infusion of chemotherapy. CR was defined as no emesis and no rescue medications. Assessment of antiemetic response (vomiting and nausea) was done as per criteria of Jones *et al* [13] as follows: Control of vomiting; CR–no emetic episode, Major response–one or two emetic episodes, Minor response–three to five emetic episodes, and Failure–more than five episodes. The intensity of nausea was evaluated on a four-point scale [27] with no nausea at one end and severe nausea (+++) at the other end. The criteria adopted for control of nausea were: no nausea (0); mild nausea (+); moderate nausea (++); and severe nausea (+++) [13, 27]. Nausea severity was evaluated by using a 100 mm visual analog scale given to the patient. The 100 mm visual analog scale ranged from 0, defined as ‘no nausea’, to 100, defined as ‘the worst nausea possible.’ If patients ranked their nausea 0–5 mm, it was considered ‘no nausea’ and if ranked 6–33 mm mild nausea, 34–66 mm moderate nausea, and 67–100 mm severe nausea. These criteria were more simple and easy to understand for the patients and relatives as they have to record the parameters for nausea and vomiting for four days from day 2–5, while for the first day the patient was in the hospital, and so a healthcare professional was able to record both the frequency and intensity of nausea and vomiting episode [13].

### Safety evaluations

Adverse events (based on standard toxicity criteria) were evaluated during each treatment cycle, including type, duration and severity (mild, moderate, severe) in relation to the study drug. Physical examinations, vital signs, and clinical laboratory parameters were also assessed.

### Statistical analysis

The patients’ characteristics have been summarised and tabulated using either counts and percentages for categorical data or count, mean, median, standard error, minimum, and maximum for continuous variables. The patients were categorised according to the intensity of nausea and frequency of vomiting experienced, and the results were analysed by applying the Fisher’s exact test. Comparison between the groups for numeric variables was done using the Kruskal–Wallis test. The results of the study regarding safety, tolerability, toxicity, and response in both the groups were documented. Data were analysed using IBM SPSS statistics 20 software. All p-values were two sided, and a p-value < 0.05 was considered statistically significant.

## Results

Table 2 shows the characteristics of the patients included in the study. No statistically significant difference was noted in both the groups regarding characteristics of the patients. The median age was 52 years in PDA group and 51 years in OD group. The most common primary site was oropharynx in both the groups. All the patients tolerated both PDA and OD schedule well. No patient reported any untoward effect directly attributable to antiemetic drugs.

All the patients (100%) in both the groups were able to record and report the frequency and intensity of both nausea and vomiting, as they were explained about the procedure in detail in easy and simple language. So, good compliance was seen in both the groups about the nausea and vomiting questionnaire completion. In both the groups, none of the patients was with percutaneous endoscopic gastrostomy (PEG) tube. Table 3 shows control of chemotherapy induced vomiting in both the groups. Complete antiemetic response for first 24 hours in PDA and OD group respectively was 26 (86.7%) versus 18 (60%), which was statistically significant as p-value was less than 0.05. The differences for major response, minor response, and no response were not statistically significant in PDA versus OD group. Complete antiemetic response for delayed emesis (from day 2–5) in PDA and OD group respectively was 25 (83.3%) versus 16 (53.3%), which was statistically significant as p-value was less than 0.05. Statistically significant difference was seen in PDA group as compared to OD group in terms of overall response (OR).

Table 4 shows the control of chemotherapy induced nausea in both the groups. For first 24 hours, in acute phase, no nausea was noted in 21 (70%) versus 14 (46.6%) in PDA and OD group respectively, which was statistically not significant. The mild, moderate, and severe nausea were statistically not significant in PDA group as in comparison to OD group for first 24 hours. No nausea in delayed phase from day 2–5 in PDA and OD group respectively was 23 (76.6%) versus 13 (43.3%), which was statistically significant (p < 0.05). No statistically significant difference was seen for mild, moderate, and severe nausea in the PDA group as compared to OD group.

**Figure 1.**
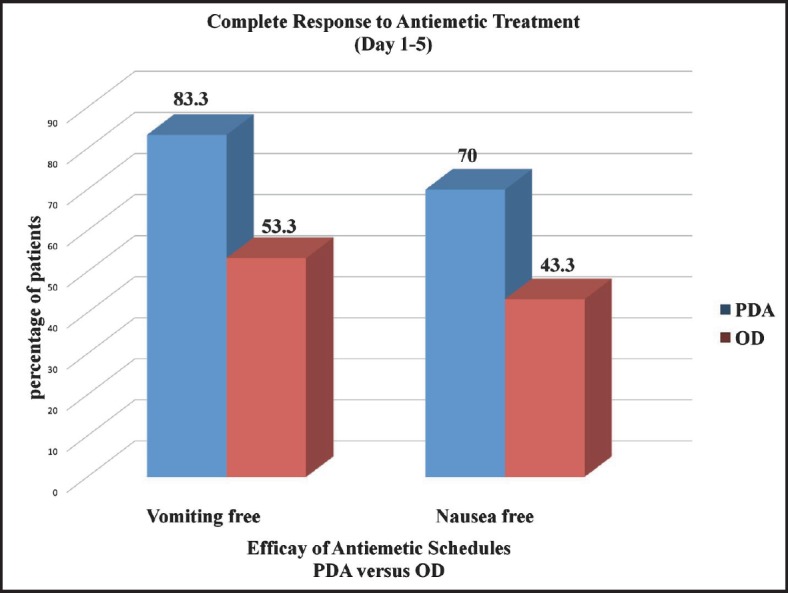
Graph showing CR to antiemetic treatment with PDA versus OD schedule.

The CR to both acute and delayed emesis (no vomiting from day 1–5) in PDA and OD group respectively was 25 (83.3%) versus 16 (53.3%) with p-value < 0.05, which is shown in Figure 1. The CR to nausea (no nausea from day 1–5) in PDA and OD group respectively was 21 (70%) versus 13 (43.3%) with p-value < 0.05, which is shown in Figure 1.

Both the antiemetic schedules were well tolerated by the patients without significant treatment-related toxicity.

## Discussion

In this open labeled, prospective CINV prophylaxis study in Indian patients with head and neck cancer, triplet antiemetic therapy with aprepitant, palonosetron, and dexamethasone, was associated with high CR (complete response) rates over doublet antiemetic therapy with ondansetron and dexamethasone, in both acute as well as delayed phases of CINV after administration of MEC with docetaxel, carboplatin, and 5-FU. In accordance with the National Comprehensive Cancer Network (NCCN) 2014 guidelines, in the present study, palonosetron was not followed by 5-HT3 antagonist post day one; only dexamethasone and aprepitant were administered post day one in PDA schedule as per the dosage prescribed by NCCN. Similarly, ondansetron and dexamethasone were administered in OD schedule in acute as well as delayed phases as per the NCCN antiemetic guidelines.[28] According to NCCN guidelines, antiemetic regimens should be chosen based on the drug with highest emetic risk. The combination chemotherapy consisting of docetaxel, carboplatin, and 5-FU which we used here is a moderately emetogenic regimen as carboplatin is a moderately emetogenic drug [28].

The data available are very limited regarding the CINV risk and antiemetic regimen efficacy in Indian patients after administration of the MEC regimen in head and neck cancer. The present study compares palonosetron and aprepitant containing group directly to ondansetron containing group. It is the first kind of this study in the Indian population. For standard antiemetic combination of ondansetron and dexamethasone (OD schedule), the protection remains largely limited to acute phase with little or no effect over delayed phase of CINV. For the treatment of chemotherapy-induced emesis, the U.S. Food and Drug Administration approved two novel agents, palonosetron, a longer-acting serotonin antagonist, and aprepitant, a neurokinin-1 antagonist [13]. For a triplet chemotherapy regimen, there is no established antiemetic schedule.

In the present study, the PDA group was well tolerated and yielded statistically significant results as compared to OD schedule in controlling CINV in acute (86.7% versus 60%) as well as delayed phase (83.3% versus 53.3%). Palonosetron is a newer, potent, and selective second generation 5-HT3 receptor antagonist and is useful for the prevention of delayed CINV in patients receiving MEC [13]. Phase III trials have demonstrated that a single dose of palonosetron compared with traditional 5-HT3 receptor antagonists is more effective in preventing acute CINV, and it also exhibits prolonged efficacy to provide significantly better protection from CINV in the delayed and overall phases as shown in a meta-analysis by Botrel *et al* in 2010 [29]. Less nausea was noted in both acute (RR = 0.86) and delayed (RR = 0.82) phases among patients in palonosetron group [28]. They also had less acute vomiting (RR 0.76) and delayed vomiting (RR = 0.76) [29]. Hajdenberg *et al* demonstrated CR rates of 84% in acute phase and 59% in delayed phase CINV for palonosetron, which is similar to our study [30].

In the present study, dexamethasone was administered in both the groups as per the dosage schedule of NCCN 2014. It has been shown that the antiemetic potential of palonosetron is significantly increased when combined with dexamethasone [28]. On combining aprepitant to palonosetron and dexamethasone in PDA group, significant improvement was noted in delayed phases of CINV. Aprepitant, a recently approved drug, antagonises the effect of substance P on neurokinin type 1 receptors [22, 23]. It has shown promising results in controlling both phases of CINV [22, 23]. The addition of aprepitant, to an antiemetic combination improves control of emesis by a further 15–20% and improves late phase symptoms (>24 hours after chemotherapy) [22, 23]. Aprepitant inhibits CYP3A4 and in turn inhibits the metabolism of dexamethasone. So, to maintain dexamethasone at the prescribed blood level in the presence of aprepitant, the dose of dexamethasone has to be reduced by 50% [31]. Although a previous population pharmacokinetic study of dexamethasone combined with aprepitant supported the validity of this dose reduction of dexamethasone, there has been no full pharmacokinetic study of dexamethasone and aprepitant in cancer patients who receive emetogenic cancer chemotherapy [32, 33].

In the present study, the proportion of patients who remained emesis free was significantly higher with the aprepitant and palonosetron containing regimen than with the active control (83.3% versus 53.3%) which is similar to the CR reported in medical literature (35.7–93% versus 5.6–66.4% respectively) [34–38]. According to Warr *et al*, the proportion of patients who remained emesis free was significantly higher with the aprepitant containing regimen than with the active control (70.2–82.8% versus 38.6–66.4% respectively) [34]. A KCOG-G1003 reported emesis free rate in overall phase, acute phase, and delayed phase as 54.2%, 87.5%, and 56.3% respectively. These results for emesis control for nausea and vomiting are similar to our study [39].

The present study has shown nausea free rate of 70% in aprepitant containing arm, which is comparable to 59.9% reported by Longo *et al* and 66.7% by Gore *et al* respectively [35, 37]. Similar results for nausea control have been reported in other studies where aprepitant or palonosetron has been used [40–43]. Rojas *et al* reported a study on molecular mechanisms of 5-HT(3) and NK(1) receptor antagonists in prevention of emesis [43]. Aprepitant not only reduced acute emesis but also helped in the reduction of delayed emesis [43].

Both the groups are cost effective as per the World Health Organisation (WHO) cost effective and strategic planning guidelines. In an Indian scenario, the cost of PDA group is much more than that of the OD group. But, as the PDA group is more effective in controlling CINV, PDA group is affordable. Recent research for management of chemotherapy-induced nausea and vomiting has focused on optimisation of palonosetron and aprepitant-based antiemetic regimens, particularly in combination with steroids. These have shown complete emesis control varying from 35.7–93% in various studies [34–43]. The study was limited by small sample size which further necessitates the need of a large randomised study trial to confirm the benefits of MEC drug combination regimens.

## Conclusion

In conclusion, the present study has shown that an antiemetic schedule comprising of palonosetron, aprepitant, and dexamethasone was better than the combination of ondansetron and dexamethasone in controlling cancer CINV in the acute as well as delayed phases post MEC in head and neck cancer. Both the antiemetic combinations were well tolerated by patients with no augmentation of any adverse drug interaction with co-prescribed medications in patients receiving chemotherapy. Future research should be focused on a large randomised trial including a larger number of patients to consolidate the benefits.

## Conflicts of interest

The authors have no conflicts of interest to declare.

## Figures and Tables

**Table 1.** Antiemetic schedules given in PDA and OD groups (n = 60).

	PDA schedule (palonosetron+ dexamethasone + dprepitant)	OD schedule (ondansetron+ dexamethasone)
**Day 1**	**Half an hour before chemotherapy (CT)** Injection palonosetron 0.25 mg IV Injection dexamethasone 12 mg IV **After CT** Capsule aprepitant 125 mg orally OD	**Half an hour before chemotherapy** Injection ondansetron 16 mg IV Injection dexamethasone 12 mg IV **After CT** Tablet ondansetron 8 mg BD
**Day 2**	Tablet dexamethasone 8 mg BD Capsule aprepitant 80 mg OD	Tablet dexamethasone 8 mg BD Tablet ondansetron 8 mg BD
**Day 3**	Tablet dexamethasone 8 mg BD Capsule aprepitant 80 mg OD	Tablet dexamethasone 8 mg BD Tablet ondansetron 8 mg BD

**Table 2.** Patient characteristics for both the groups included in the study [n(%)].

Characteristics		PDA Group	OD Group
**Age (years)**	Range	36–70	34–69
	Median	52	51
**Gender**	Male	29 (96.7)	23 (76.7)
	Female	1 (3.3)	7 (23.3)
**Karnofsky Performance Status**	≥80	24 (80)	25 (83.3)
	<80	6 (20)	5 (16.7)
**Tumour stage (AJCC)**	III	12 (40)	11 (36.7)
	IV	18 (60)	19 (63.3)
**Primary site**	Oral cavity	3 (10)	5 (16.7)
	Oropharynx	18 (60)	15 (50)
	Nasopharynx	1 (3.3)	0
	Larynx	5 (16.7)	6 (20)
	Hypopharynx	3 (10)	4 (13.3)

**Table 3.** Comparison of chemotherapy induced vomiting control in both the groups as PDA versus OD group [n(%)].

	Antiemetic response Jones *et al*^10^	Acute phase (day 1) No. (%)	Delayed phase (day 2–5)^*^ No. (%)	Overall Response (Day 1–5) No. (%)
**Complete response**	No emetic episode	26 (86.7%) versus	25 (83.3%) versus	25 (83.3%) versus
		18 (60%)	16 (53.3%)	16 (53.3%)
		p = 0.01, significant	p = 0.01, significant	p = 0.01, significant
**Major response**	1–2 emetic episodes	4 (13.3%) versus	5 (16.6%) versus	4 (13.3%) versus
		9 (30%)	8 (26.6%)	6 (20%)
		p = 0.12, NS^**^	p = 0.35, NS^**^	p = 0.49, NS
**Minor response**	3–5 emetic episodes	0 (0%) versus	0 (0%) versus	01 (3.33%) versus
		2 (6.6%)	4 (13.3%)	5 (16.7%)
		p = 0.15, NS^**^	p = 0.03, significant	p = 0.08, NS^**^
**No response**	>5 emetic episodes	0 (0%) versus 1 (3.3%)	0 (0%) versus 2 (6.6%)	0 (0%) versus 3 (10%)
		p = 0.31, NS^**^	p = 0.15, NS^**^	p = 0.07, NS^**^

* Delayed response is graded depending upon the worst observation from day 2–5.

** NS = Not significant

**Table 4.** Comparison of chemotherapy induced nausea control in both the groups as PDA versus OD group [n(%)].

Schedule	Acute phase (day 1) No. (%)	Delayed phase (day 2–5)^*^ No. (%)	Overall response (day 1–5) No. (%)
**No nausea**	21 (70%) versus	23 (76.6%) versus	21 (70%) versus
	14 (46.6%)	13 (43.3%)	13 (43.3%)
	p = 0.06, NS^**^	p < 0.05, significant	p = 0.04, significant
**Mild nausea (+)**	6 (20%) versus	6 (20%) versus	6 (20%) versus
	9 (30%)	9 (30%)	9 (30%)
	p = 0.37, NS^**^	p = 0.37, NS^**^	p = 0.37, NS^**^
**Moderate nausea (++)**	3 (10%) versus	1 (3.3%) versus	2 (6.7%) versus
	5 (16.6%)	6 (20%)	6 (20%)
	p = 0.95, NS^**^	p = 0.44, NS^**^	p = 0.13, NS^**^
**Severe nausea (+++)**	0 versus 2 (6.6%)	0 versus 2 (6.6%)	1 (3.3%) versus
	p = 0.15, NS^**^	p = 0.15, NS^**^	2 (6.6%)
			p = 0.55, NS^**^

* Delayed response is graded depending upon the worst response from day 2–5.

** NS = Not significant
